# Identifying Cross-Scale Associations between Radiomic and Pathomic Signatures of Non-Small Cell Lung Cancer Subtypes: Preliminary Results

**DOI:** 10.3390/cancers12123663

**Published:** 2020-12-07

**Authors:** Charlems Alvarez-Jimenez, Alvaro A. Sandino, Prateek Prasanna, Amit Gupta, Satish E. Viswanath, Eduardo Romero

**Affiliations:** 1 Computer Imaging and Medical Application Laboratory, Universidad Nacional de Colombia, Bogotá 111321, Colombia; calvarezj@unal.edu.co (C.A.-J.); asandino@unal.edu.co (A.A.S.); 2 Department of Biomedical Engineering, Case Western Reserve University, Cleveland, OH 44106, USA; satish.viswanath@case.edu; 3 Department of Biomedical Informatics, Stony Brook University, Stony Brook, NY 11794, USA; prateek.prasanna@stonybrook.edu; 4 Department of Radiology, University Hospitals Cleveland Medical Center, Cleveland, OH 44106, USA; amit.gupta@uhhospitals.org

**Keywords:** lung cancer, cell density, radiomics, pathomics, correlation, association, CT, digital pathology

## Abstract

**Simple Summary:**

This work presents initial results for differentiating two major non-small cell lung cancer (NSCLC) subtypes by exploring cross-scale associations between Computed Tomography (CT) images and corresponding digitized pathology images. The analysis comprised three phases, (i) a multi-resolution cell density quantification to identify discriminant pathomic patterns, (ii) radiomic characterization of CT images by using Haralick descriptors, and (iii) quantitative correlation analysis between the multi-modal features to identify potential associations between them. This analysis was carried out using publicly available databases, two digitized pathology and two radiology cohorts. Preliminary but significant cross-scale associations were identified between cell density statistics and CT intensity values using matched specimens, which were used to significantly improve the overall discriminatory performance of radiomic features in differentiating NSCLC subtypes.

**Abstract:**

(1) Background: Despite the complementarity between radiology and histopathology, both from a diagnostic and a prognostic perspective, quantitative analyses of these modalities are usually performed in disconnected silos. This work presents initial results for differentiating two major non-small cell lung cancer (NSCLC) subtypes by exploring cross-scale associations between Computed Tomography (CT) images and corresponding digitized pathology images. (2) Methods: The analysis comprised three phases, (i) a multi-resolution cell density quantification to identify discriminant pathomic patterns for differentiating adenocarcinoma (ADC) and squamous cell carcinoma (SCC), (ii) radiomic characterization of CT images by using Haralick descriptors to quantify tumor textural heterogeneity as represented by gray-level co-occurrences to discriminate the two pathological subtypes, and (iii) quantitative correlation analysis between the multi-modal features to identify potential associations between them. This analysis was carried out using two publicly available digitized pathology databases (117 cases from TCGA and 54 cases from CPTAC) and a public radiological collection of CT images (101 cases from NSCLC-R). (3) Results: The top-ranked cell density pathomic features from the histopathology analysis were correlation, contrast, homogeneity, sum of entropy and difference of variance; which yielded a cross-validated AUC of 0.72 ± 0.02 on the training set (CPTAC) and hold-out validation AUC of 0.77 on the testing set (TCGA). Top-ranked co-occurrence radiomic features within NSCLC-R were contrast, correlation and sum of entropy which yielded a cross-validated AUC of 0.72 ± 0.01. Preliminary but significant cross-scale associations were identified between cell density statistics and CT intensity values using matched specimens available in the TCGA cohort, which were used to significantly improve the overall discriminatory performance of radiomic features in differentiating NSCLC subtypes (AUC = 0.78 ± 0.01). (4) Conclusions: Initial results suggest that cross-scale associations may exist between digital pathology and CT imaging which can be used to identify relevant radiomic and histopathology features to accurately distinguish lung adenocarcinomas from squamous cell carcinomas.

## 1. Introduction

According to the World Health Organization (WHO), lung cancer was the most common cancer diagnosed worldwide in 2018 [1]. Between 80% to 85% of lung cancers are non-small cell lung cancer (NSCLC), and the most prevalent subtypes are adenocarcinoma (ADC) and squamous cell carcinoma (SCC) [2,3]. ADC is the most commonly diagnosed type of lung cancer and frequently occurs along the outer periphery of the lung [4,5]. SCC comprises 25–30% of lung cancer cases and usually occurs in the central portion of the lung as well as being considered more aggressive than ADC [4]. Other clinicopathologic differences are highlighted in Table S1. Available treatment options include conventional chemotherapy and targeted therapies, and typically differ for ADC and SCC [6,7]. Therefore, early characterization of lung cancer subtypes can be highly relevant for guiding the therapeutic regimen (curative or palliative [8]). The key modalities in the lung cancer clinical protocol remain standard-of-care computed tomography (CT) imaging (acquired at diagnosis) as well as tissue biopsy specimens (acquired for disease confirmation); both typically requiring subjective expert evaluation in the clinical workflow [9].

Towards developing more objective measurements and approaches for these modalities, there has been a recent focus on developing *radiomic* and *pathomic* strategies. ‘Radiomics’ refers to the automated extraction of quantitative information associated with manifestations of pathologies on a radiographic imaging scale, specifically imaging attributes that are not visually identifiable [10,11]. Radiomic attributes have been demonstrated to be correlated with disease risk in prostate [12,13,14], breast [15,16,17], lung [9,18,19,20], brain [21,22], colorectal [23,24,25], renal cell carcinoma [26], and head and neck [27,28] cancers. Specifically, radiomic approaches to discriminate ADC from SCC on CT imaging primarily build upon morphometric and textural features [9,29,30,31,32]. ’Pathomics’ involves computational characterization of complex patterns on digitized histopathology slides [33,34], whole slide images (WSI), to describe diverse phenotypic characteristics of cancer and patient risk in a variety of organs [35,36], including lung [37,38]. On hematoxylin and eosin (H&E) digitized slides of lung cancer specimens, several pathomics strategies based on topological [39], morphological [40], and convolutional neural networks (CNNs) strategies, have been used to capture complex patterns characterizing different pathological subtypes [41,42]. As CT and digitized pathology offer complementary sources of information about the tumor in vivo, a natural question is whether these radiomics and pathomics features might be connected.

There have been recent efforts on identifying cross-scale associations between radiology and pathology scales in different cancers [43,44,45,46,47,48], albeit not specifically for the question of distinguishing ADC from SCC. While ADC is characterized by malign glandular structures and mucin, it grows following different patterns, including acinar, lepidic, papillary, micropapillary and solid [49,50,51]. By contrast, SCC infiltrates the peri-bronchial tissue and grows resembling a cauliflower [50,51]. An optimal computational strategy to differentiate these two lung cancer subtypes on CT should therefore be designed to exploit the underlying architectural differences in the internal organization of cell populations on pathology images.

This study presents initial results for a novel pathomic-radiomic association approach, i.e., identifying pathomic features from digitized histopathology that potentially reflect the tissue composition basis of radiomic descriptors from CT, towards improving the understanding and ability to discriminate ADC from SCC. The underlying hypothesis is that pathomics features that can differentiate ADC from SCC could be used to determine a pathologic or morphologic basis for radiomic expression patterns on CT. The presented approach will involve first evaluating co-occurrence-based texture representations of (a) cellular density on pathology, and (b) CT intensity values on imaging; for differentiating ADC from SCC using each modality independently. Finally, a preliminary exploration of the cross-scale relationships between the radiomic and pathomic representations will be tested by systematically comparing correlations between them.

## 2. Materials and Methods

### 2.1. Digitized Pathology Data

A total of 171 patients were included in this study from two public datasets: (i) The National Cancer Institute’s Clinical Proteomic Tumor Analysis Consortium (CPTAC) [52,53], and (ii) The Cancer Genome Atlas (TCGA) and Genomic Data Commons Data Portal [54]. The CPTAC dataset comprised 117 studies while 54 studies were obtained from TCGA. Here, the CPTAC database was used for training while hold-out testing was performed with the TCGA database. Each study had at least one whole slide specimen available that had been stained with hematoxylin and eosin (H&E) and digitized in the SVS format. Table 1 summarizes the scanning parameters and the study population curated for use in this study, based on information reported in the public databases.

### 2.2. Computed Tomography Imaging Data

A total of 146 patients were included in this investigation from two public datasets: (i) NSCLC-Radiomics, Maastro Lung1 dataset (NSCLC-R) [55] whose cases were collected between 2004 and 2014 at the Maastro Clinic in The Netherlands, and (ii) The Cancer Genome Atlas (TCGA) and Genomic Data Commons Data Portal [54] with cases captured between 1988 and 2005 from many sites all over the world. For each study, a computed tomography (CT) scan is available, for which the scanner, imaging, and population parameters are summarized in Table 2. Note that the CT scans in the TCGA dataset correspond to the same patients for whom WSI images were curated (Section 2.1). Pathologic subtype diagnosis for all CT scans was determined based on information reported in each database.

The NSCLC-R dataset included the associated tumor expert annotations, while for the TCGA dataset, manual delineation of the primary gross tumor volume was performed by an expert radiologist (AG). Crucially, there were important differences between the two databases; while NSCLC-R was quite homogeneous, TCGA studies were highly variable in terms of imaging details. Specifically, NSCLC-R studies were acquired with two different scanner manufacturers but with a similar in-plane resolution, slice thickness, field of view, and patient position across all studies. By contrast, most of these imaging parameters are highly variable in TCGA studies because of the 17-year period of acquisition and concomitant inter-site differences in voxel size, contrast, and imaging sequence. Consequently, classification experiments were performed with the NSCLC-R database alone, while the TCGA database was used to establish correlations between histopathological and radiological attributes.

### 2.3. Computing Cellular Density Map on Histopathology Slides

**Figure 1:**
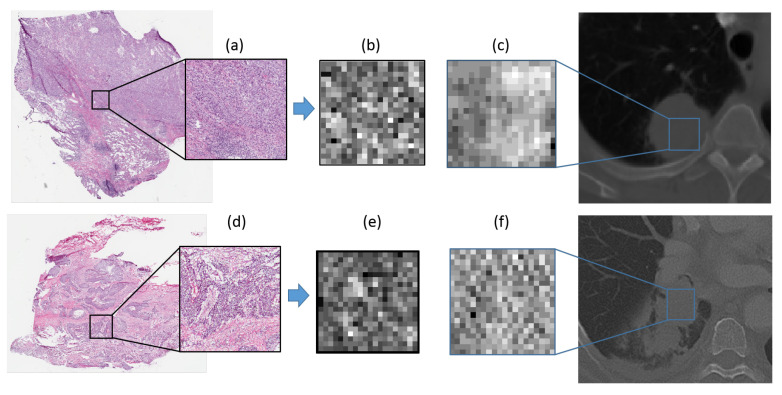
Estimating cellular density on histopathology slides: (**a**,**d**) illustrate a patch from each WSI, (**b**,**e**) show the associated cell density map, and (**c**,**f**) depict a corresponding tumor patch from CT. The top row shows a representative ADC while the second corresponds to a SCC study.

A patch-based strategy was employed to analyze each WSI. First, a histopathology grid with patches of 1 mm^2^ was constructed (equivalent to 2000 × 2000 pixels for a 20× magnification), which was in turn divided into tiles of 50 μm^2^. The cellular density was estimated in each of these tiles by segmenting nuclei [56] and assigning a gray level to each of these tiles based on the number of nuclei estimated in each tile. This resulted in a spatial map of the cellular density on the digitized histopathology, termed the *cell density map*. Figure 1b,e illustrates the associated cell density map for the histopathology patches in Figure 1a,d. In addition, Figure 1c,f depicts the tumor patch from the CT scan of the same patient. The top row shows a representative ADC while the second corresponds to a SCC study.

### 2.4. Pathomics Feature Extraction From Cellular Density Map

**Figure 2:**
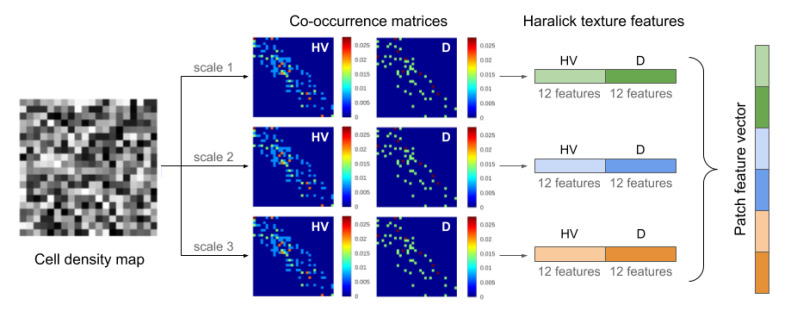
Multi-scale texture analysis of cellular density on digitized pathology to compute pathomic features: the cell density map of a particular histopathology patch is used to compute two co-occurrence matrices (one along the horizontal-vertical direction and one along the diagonal direction) per scale. These are then utilized to compute 12 Haralick texture features per co-occurrence matrix, from which statistics are computed to result in 360 pathomic descriptors.

Although SCC is characterized by an accelerated cell growth compared to ADC [57] (and thus an increased cell density), first order statistics of the cell density map by themselves may not suffice to differentiate between these two lung cancer subtypes. Therefore, the spatial distribution of cell density on each WSI was computed using Haralick features extracted at three different scales, as illustrated in Figure 2. From each matrix of cell density values, two co-occurrence matrices were calculated per scale (horizontal-vertical and diagonal directions), to estimate how often a particular cell density value co-occurred with its neighbors. For each co-occurrence matrix, 12 Haralick texture features [58] were computed to obtain a feature vector of 72 descriptors per patch. To obtain a feature vector per case, considering that each WSI is represented by a different number of patches, five statistics (mean, median, variance, kurtosis, and skewness) were computed across the entire set of patches and each of the 72 descriptors. Therefore, a 360 × 1 pathomic feature vector was used to describe each study.

### 2.5. Radiomics Feature Extraction from CT Images

**Figure 3:**
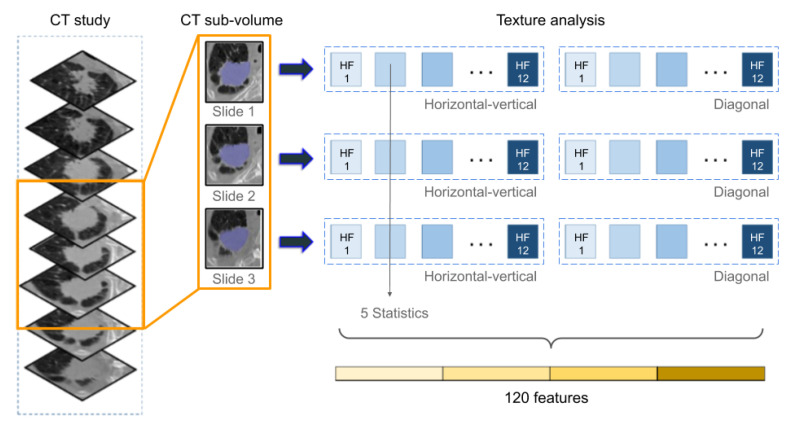
Texture analysis in CT to compute radiomic features: For each CT study, a sub-volume is defined as the largest annotated 2D section of the tumor together with two adjacent slices. For each sub-volume, co-occurrence matrices are computed based on CT image intensity values, based on which 12 Haralick features (HF) are calculated. Finally, five statistics are computed per Haralick feature to yield 120 radiomic descriptors.

For radiomic feature extraction, the region of interest (ROI) was defined as a sub-volume of the largest annotated 2D tumor section together with the two adjacent Sections (3 consecutive 2D sections in total), as illustrated in Figure 3. This was the smallest sub-volume consistently available for all patients. Per section, 12 Haralick textural features were computed from two co-occurrence matrices (horizontal-vertical and diagonal directions) that had been computed based on CT image intensity values. Five statistics were then calculated to characterize the texture feature distribution in the CT sub-volume, resulting in 120 radiomic texture descriptors per study.

### 2.6. Experimental Design

#### 2.6.1. Experiment 1: Identifying Pathomics Features to Differentiate ADC from SCC

To analyze the spatial distribution of cell density values across histopathology tiles, normalized co-occurrence matrices from cellular density maps were visualized as heatmaps where red values corresponded to higher likelihood of co-occurrence. Two sets of pathomics features were evaluated for differentiating the two non-small cell lung cancer subtypes, one using a set of Haralick-based pathomic descriptors (from the co-occurrence matrices, denoted *F^PH^*) as well as using the five statistics (mean, median, standard deviation, skewness and kurtosis) computed from the cell density distribution (denoted *F^PS^*). The CPTAC database was used as the training cohort to identify and evaluate these feature sets through a randomized 10-fold cross validation setting, with further validation using the TCGA database in a hold-out setting.

#### 2.6.2. Experiment 2: Identifying Radiomics Features to Differentiate ADC from SCC

This experiment was carried out to assess the ability of radiomics features to discriminate ADC from SCC. Two classification tests were performed in the NSCLC-R dataset under a randomized 10-fold cross validation scheme, first with the five statistics computed from image intensities within the tumor ROI (denoted *F^RS^*), and next using a set of Haralick-based radiomic descriptors (denoted *F^RH^*).

#### 2.6.3. Experiment 3: Exploratory Identification of Cross-Scale Pathomic-Radiomic Associations

This experiment was conducted to preliminarily identify and evaluate pathomic-radiomic associations, i.e., a set of correlated pathomics and radiomics features. The first test evaluated the correlation between cell density attributes and image intensities using the TCGA cohort (where both radiology and histopathology images were available), i.e., between *F^PS^* and *F^RS^*. Next, Haralick-based radiomic descriptors were limited to the specific feature types that had been identified as relevant in Experiment 1 (i.e., at the pathologic scale). This subset was then evaluated to discriminate ADC and SCC at the radiological scale and compared in performance to the descriptors identified in Experiment 2. This test was carried out using the NSCLC-R dataset under a 10-fold cross validation scheme.

### 2.7. Statistical Analysis

The selection of pathomics and radiomics features was performed as follows: (i) feature normalization was applied to ensure that all extracted descriptors were within the same range of values by subtracting the mean and dividing by the standard deviation, and (ii) discriminant features were selected using a combination of significance and correlation testing, to ensure feature relevance while removing potentially redundant and non-informative descriptors.

Each set of top-ranked radiomic and pathomic features were individually evaluated by a classification task at discriminating ADC from SCC using a support vector machine (SVM) model with a linear kernel. Model performance was quantified in terms of the area-under-receiver-operator-curve (AUC), together with confidence intervals.

Correlations were statistically evaluated as follows: (i) the correlation coefficient and the associated *p*-values were computed for each pair of *F^PS^* and *F^RS^* features using Spearman’s rank correlation test (to ensure robustness to outliers while quantifying monotonicity [59]), followed by (ii) identifying significant correlations using False Discovery Rate (FDR) correction for multiple hypotheses testing [60], with statistical significance when FDR ≤ 0.01. This threshold was selected to reflect the preliminary nature of this study.

Additional experiments were conducted to evaluate the robustness of the different feature sets as well as the cross-scale associations. Top-ranked pathomics features identified in Experiment 1 were assessed for statistical significance at different tile sizes (between 50 μm^2^, 33.3 μm^2^, and 25 μm^2^) via Wilcoxon ranksum testing while comparing ADC vs SCC in the CPTAC database. Similarly, top-ranked radiomic features from Experiment 2 were assessed for differences between the two manufacturers (Siemens, CMS) in the NSCLC-R cohort via Wilcoxon ranksum testing. Finally, as the number of patches available from each WSI varied by dataset (6–67 patches/dataset), trends in Spearman’s rank correlation testing between pathomic-radiomic features were re-evaluated within 2 subsets: for cases with <15 patches/study and for cases with ≥15 patches/study.

## 3. Results

### 3.1. Experiment 1: Identifying Pathomic Features to Differentiate ADC from SCC

**Figure 4:**
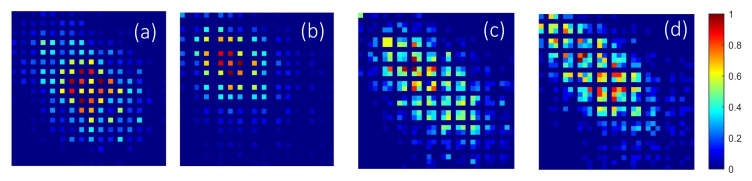
Representative normalized co-occurrence matrices computed in horizontal-vertical directions from cell density maps: (**a**,**b**) correspond to ADC studies, and (**c**,**d**) to SCC studies. Note the more homogeneous pattern in SCC with smoother variation in cooccurrences compared to ADC.

**Figure 5:**
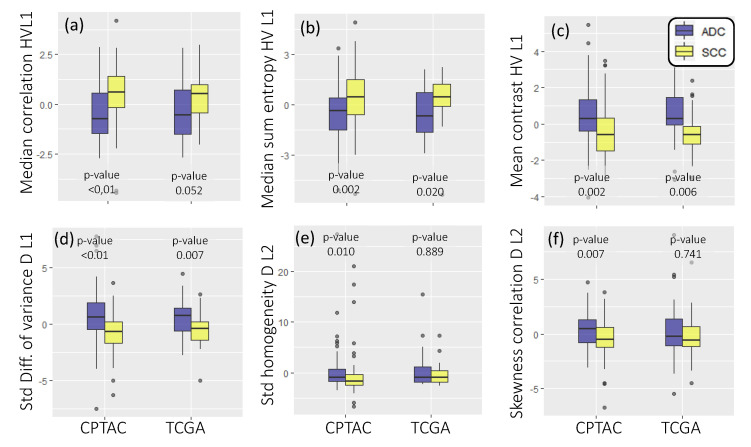
Box plots (**a**–**f**) of the six top-ranked pathomics features together with their *p*-value from Wilcoxon ranksum testing (unadjusted), when comparing ADC (blue) to SCC (yellow). Box plots are presented for both histopathological datasets considered, CPTAC (training) and TCGA (testing).

Heat maps of normalized co-occurrences computed from cell density maps illustrate that ADC (Figure 4a,b) show a higher and more concentrated co-occurrences of cell density values between adjacent tiles. The opposite trend is observed in SCC (Figure 4c,d), i.e., co-ocurrences are more distributed with a smoother changes in cell density values between adjacent tiles. Correspondingly, Figure 5a–f shows the box and whisker plots of the top-ranked Haralick-based pathomics features that comprise *F^PH^*, together with their *p*-values from Wilcoxon ranksum testing between ADC (blue) and SCC (yellow); in both TCGA and CPTAC cohorts.

Using the six top-ranked pathomics features in *F^PH^*, the SVM classifier with a linear kernel resulted in a training AUC of 0.72 ± 0.02 (95% confidence interval: 0.65–0.77) and a testing AUC of 0.77. This corresponded to an accuracy of 0.69 ± 0.03 (95% confidence interval: 0.62–0.74) in the training set and a 0.74 accuracy in the testing set (0.72 sensitivity, 0.76 specificity). By contrast, when considering $F^{PS}$, the linear SVM yielded a significantly lower training AUC of 0.62 ± 0.02 (CPTAC, 95% confidence interval: 0.55–0.68) and a testing AUC of 0.34 (TCGA).

Figure S1 shows the box plots for each of the three different tile sizes: 50 μm^2^, 33.3 μm^2^ and 25 μm^2^. These boxplots demonstrate no significant differences (all *p* > 0.05, Wilcoxon ranksum test) in any of the top-ranked Haralick-based pathomics features between tile sizes.

### 3.2. Experiment 2: Identifying Radiomic Features to Differentiate ADC from SCC

**Figure 6:**
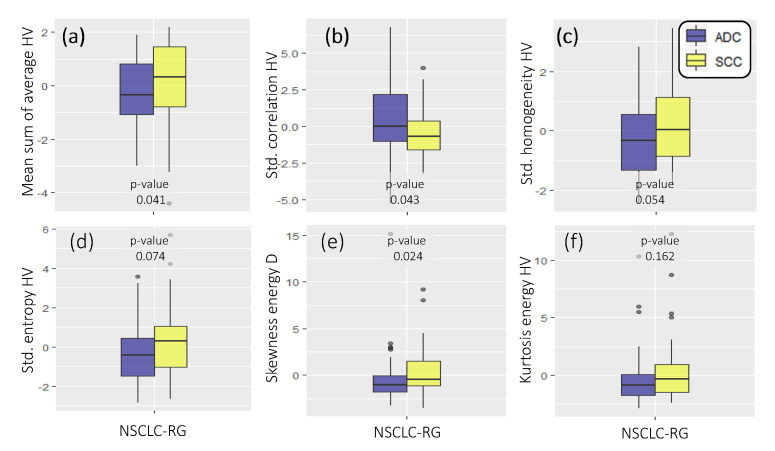
Box plots (**a**–**f**) of the top-ranked radiomics features together with their *p*-value from Wilcoxon ranksum testing (unadjusted), when comparing ADC (blue) to SCC (yellow) non-small cell lung cancer subtypes in the NSCLC-R dataset.

Figure 6a–f shows the box and whisker plots of the top-ranked radiomics descriptors that comprise $F^{RH}$ identified via cross-validation in the NSCLC-R dataset, together with their *p*-values in Wilcoxon ranksum testing between ADC (blue) and SCC (yellow). Using this set of discriminant radiomics descriptors, the SVM model yielded a cross-validated training AUC of 0.72 ± 0.01 (95% confidence interval: 0.65–0.77). This corresponded to an accuracy of 0.69 ± 0.01 (95% confidence interval: 0.61–0.74) in the NSCLC-R database, a sensitivity of 0.67 ± 0.01 (95% confidence interval: 0.59–0.73), and a specificity of 0.71 ± 0.02 (95% confidence interval: 0.63–0.76). By comparison, $F^{RS}$ yielded a significantly lower cross-validated AUC of 0.51 ± 0.04 (95% confidence interval: 0.43–0.58).

Figure S2 shows the box plots comparing top-ranked radiomic features between Siemens and CMS scans, demonstrating no significant differences in any of the top-ranked Haralick-based radiomic features between the manufacturers.

### 3.3. Experiment 3: Exploratory Identification of Pathomic-Radiomic Associations for Differentiating ADC from SCC

**Figure 7:**
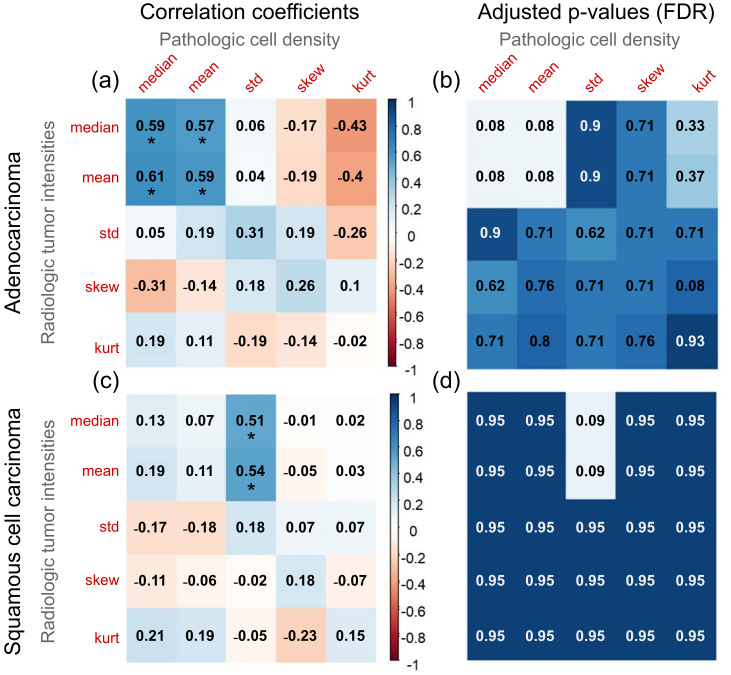
Heatmap visualizations for (**a**,**c**) Spearman’s rank correlation coefficients between pathologic cell density and radiologic intensity statistics (red = negative correlation, blue = positive correlation). (**b**,**d**) similarly visualized adjusted *p*-values for each pair of features as a heatmap, after False Discovery Rate (FDR) correction for multiple hypotheses testing.

**Figure 8:**
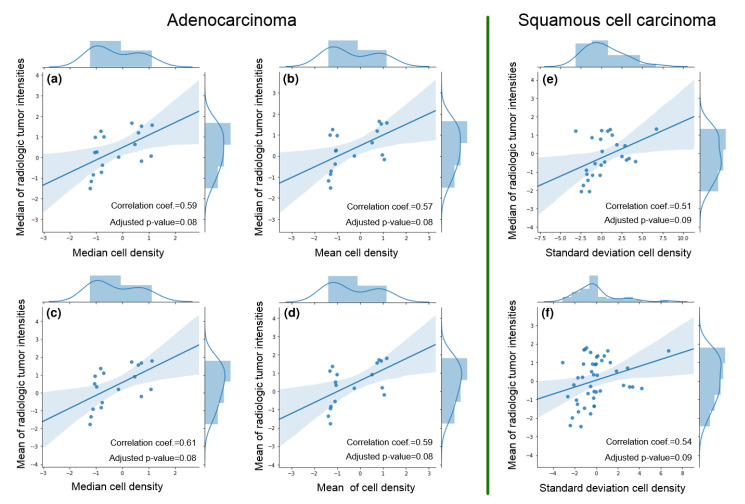
Scatter plots of six pairs of correlated features identified via pathomic-radiomic association for (**a**–**d**) ADC stuides, and (**e**,**f**) SCC studies. Also noted are correlated coefficients as well as FDR-adjusted *p*-values. The histogram of pathomic feature are plotted along the X-axes of each plot, while radiomic features are plotted along Y-axes.

Figure 7 visualizes the correlation coefficients between $F^{PS}$ and $F^{RS}$ as well as adjusted *p*-values (after FDR correction) as heatmaps; for ADC and SCC separately. As observed in Figure 7a,c, six pairs of features showed significant cross-scale associations with correlation coefficients between 0.51 and 0.61 (denoted by *, adjusted *p*-values after FDR correction in Figure 7b,d). To further evaluate these relationships, Figure 8 illustrates scatter plots for each of these six pairs of features (pathomics features on X-axes, radiomics features on Y-axes) with corresponding histograms along the border of each plot.

Figure S3 additionally illustrates the correlation coefficients for two sub-groups: cases with <15 (top) and ≥15 (bottom) patches/dataset. While ADC shows a similar trend in correlation coefficients between Figure 7a and Figure S3a,b, SCC trends are only largely consistent between Figure 7c and Figure S3b but Figure S3c is slightly different (likely due to the fewer number of cases available in this sub-group).

**Figure 9:**
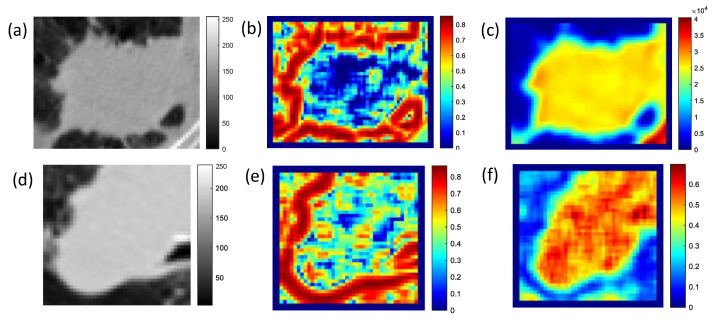
Top-ranked radiomic descriptors identified based on pathomic-radiomic associations and evaluated in Experiment 3, visualized as a heatmap (where red corresponds to over-expression): (**a**,**d**) Tumor on CT image, (**b**,**e**) Haralick correlation, (**c**,**f**) Haralick homogeneity for a representative ADC study (top row) as well as a representative SCC study (bottom row).

When limiting $F^{RH}$ to the feature types identified in Experiment 1, classification performance significantly improved to an AUC of 0.78 ± 0.01 (95% confidence interval: 0.70–0.82), compared to Experiment 2. Figure 9 depicts the top-ranked radiomic features identified in this experiment as heatmaps (Haralick correlation and homogeneity) for representative ADC and SCC lung tumors.

## 4. Discussion

The association between certain spatial patterns of sub-visual information on radiographic or pathologic images with disease outcomes [10,20,33,34] has seen wide evaluation using *radiomics* and *pathomics* approaches. In this work, we presented initial results for evaluating cross-scale associations between radiomic and pathomic descriptors; these features were subsequently applied to distinguish the two major subtypes of non-small cell lung cancer, ADC and SCC. Despite the differences in scale, radiology and pathology datasets are complementary in nature, with both forming an integral component in most clinical decision making scenarios in oncology. The underlying relationship between the two scales of information is thus highly relevant to improving the understanding of the disease as well as the patient management. In this context, the goal of this work was to present preliminary results of quantitatively correlating features from these two image modalities to more accurately interrogate disease phenotypes in lung cancers.

The first part of this study was devoted to exploring how pathomics and radiomics features independently discriminated NSCLC subtypes. At both scales, using statistical measures did not yield good classification performance. By contrast, top-ranked co-occurrence-based radiomic (based on image intensities) and pathomic (based on cell density values) features provided a consistently higher performance in discriminating ADC from SCC. On histology, when compared with ADC, SCC was characterized by lower values of mean contrast, and lower values of both standard deviation of variance and homogeneity; suggesting fewer cell density variations in this type of cancer. In addition, ADC exhibited lower median correlation than SCC, suggesting SCC exhibits higher homogeneity in pathomic textures of cell density. On CT, when compared with SCC, ADC was described by lower values of standard deviation of homogeneity, entropy, and energy; indicating a markedly lower intensity variation in this NSCLC subtype. In addition, ADC exhibited higher standard deviation of correlation than SCC, suggesting poorly defined horizontal or vertical patterns in intensity co-occurrences. Top-ranked radiomics and pathomics features were also robust and maintained their statistical trends when evaluated between variations in underlying parameters or acquisition differences. These findings resonate with previous studies on lung cancer where investigations have either quantitatively described histopathological attributes [39,40], or have described visual patterns on CT [9,29,32].

The classifier performance of our radiomics and pathomics strategies were comparable to previous efforts in the literature. Linning et al. [31], Haga et al. [29] and Wu et al. [9] investigated associations between radiomic features such as tumor shape-size, intensity statistics, and texture, with histologic subtypes (ADC and SCC) and reported an AUC of 0.73, 0.72 and 0.80, respectively. Bashir et al. [30] analyzed the influence of texture and semantic descriptors; a random forest model with semantic features provided an AUC of 0.82 in an independent test set (*n* = 100). Using wavelet-based features, Zhu et al. [32] reported an AUC of 0.89 in differentiating the two classes using CT scans. At a histopathologic level, using digitized H&E slides, several pathomics strategies have studied topological [39] and morphological [40] features and reported classification results with up to an AUC of 0.95. More recently, convolutional neural networks (CNNs) have been applied to capture complex patterns characterizing different pathological entities [41,42], reporting an AUC of 0.97. While our pathomics features yielded a lower AUC overall, this may have been because our study primarily limited itself to evaluating spatial micro-patterns of cell density rather than more complex features (to ensure a similarity in formulation with radiomic features).

Preliminary pathomic-radiomic correlation analysis was conducted between cell density values (from pathology) and image intensity values (from CT scans) to identify a set of relationships between the modalities. In ADC, the means and medians of cell densities and image intensities were found to be significantly correlated. However, in SCC, CT intensity mean and median was found to be correlated with standard deviation of the cell density. While it is difficult to demonstrate a causal relationship between these features, we can hypothesize about the underlying connections between these features. For instance, homogeneous cell density (histopathology) appears to be associated with lower intensity CT values (radiology). Only a few works have similarly correlated radiology and histopathology information, and efforts have been rather limited to establishing qualitative clinical correlation. In the context of lung cancer, Ganeshan et al. [61] obtained CT images from a set of patients with NSCLC who later underwent an intravenous administration of a marker of tumor hypoxia and angiogenesis. They then assessed correlations between the CT-derived radiomic texture features and the histopathologic markers. They demonstrated radiomic features capture heterogeneity and have the potential to act as imaging correlates for tumor hypoxia and angiogenesis. Snoeckx et al. [62] studied visual pathology-radiology concordance by establishing a level of agreement between findings of radiologists and pathologists in the task of classifying pulmonary nodules into solid or subsolid phenotypes. Lederlin et al. [47] evaluated associations between qualitative CT morphological parameters (tumor shape, sphericity, location, margins and attenuation) and histomorphological growth patterns of pulmonary ADCs (lepidic, acinar, papillary, micropapillary and solid), reporting a set of specific CT features predictive of the histomorphological tumor growth patterns of pulmonary ADC. Most recently, Khorrami et al. [48] identified which radiomic patterns were associated with response and survival to immunotherapy for lung cancers, and determined that specific Gabor responses in the peritumoral region were associated with the density of tumor-infiltrating lymphocytes on diagnostic biopsy samples from these patients. Unlike these works, our study utilized similar formulations of co-occurrence features to establish potential associations between computational textural patterns on both radiology and digitized pathology. These associations were also largely consistent for each NSCLC subtype when evaluated between parameter variations. Despite the limited set of data, results suggest these associations could support clinical decisions. Such models are needed to map each modality onto the other and enrich a final radiology-pathology microscopic representation. To further demonstrate the advantage of exploiting such a pathomic-radiomic association, top-ranked co-occurrence descriptors identified at the pathologic scale were found to yield an improved performance in differentiating lung cancer subtypes when extracted at the radiologic scale.

We do acknowledge some shortcomings of our study. While we utilized large public databases, our final cohort sizes were still limited (*n* = 171 pathology speciments, *n* = 146 radiology datasets). However, we ensured our analysis was conducted as rigorously as possible with cross-validation as well as hold-out validation where possible. The radiology-pathology associations evaluated in this study only leveraged a subset of the total datasets considered, which is testament to the difficulty in collecting such matched specimens. We attempted to account for these limited numbers by appropriately adjusting the false discovery rate threshold. Finally, we only evaluated co-occurrence based features in this study rather than more complex features (e.g., architectural, topological from pathology; wavelet, gradients from radiology). This was done in order to be able to directly translate descriptors from one scale to the next as done in our pathomic-radiomic analysis experiments. Our framework may be used to study associations between an expanded suite of radiomic and pathomic measurements, as well as extended to characterizing other pathologic subtypes of lung cancers.

## 5. Conclusions

In this study, preliminary results were presented for a novel pathomic-radiomic correlation approach to interrogate cross-scale associations between cellular density and image heterogeneity across radiology and histopathology. The present investigation has demonstrated that spatial patterns of tumor heteogeneity may be shared between these modalities, as quantified via radiomics and pathomics features. Exploratory analysis showed that these associations may be further exploited to significantly improve classifier performance and quantitative characterization of lung adenocarcinoma from squamous cell carcinoma. Future work will involve validating our findings and approach on a larger cohort of data, as well as extending this approach to other cancers.

## Figures and Tables

**Table 1:** Summary of imaging parameters, demographic, and pathologic information of the whole slide image (WSI) cohort used in this study.

Parameter		CPTAC (117)	TCGA (54)
Magnification	20×	117	29
	40×	0	25
Resolution (μm∖px)	0.252	0	25
	0.494	117	0
	0.502	0	29
Gender	Male	82	23
	Female	35	31
Age at diagnosis		65.1 ± 9.3	68.6 ± 10.00
Grade	Well differentiated	8	NA
	Moderately differentiated	109	NA
	Poorly differentiated	0	NA
	Undifferentiated	0	NA
Pathologic Stage	I	58	19
	II	38	18
	III	21	12
	IV	0	3
	Discrepancy	3	2
NSCLC subtype	ADC	61	25
	SCC	56	29

**Table 2:** Summary of imaging parameters, demographics, and pathologic information of CT images used in this study.

Parameter		NSCLC-R (101)	TCGA (45)
In-plane Resolution (mm)		0.97	0.6–0.97
Slice Thickness (mm)		3	2–5
Field of view (px)		512 × 512	357–512 × 357–512
Scanner	CMS, Inc.	11	0
	Siemens	90	10
	GE Medical Systems	0	33
	Philips	0	2
Patient Position		Head First Supine (HFS)	NA
Gender	Male	64	20
	Female	37	25
Age at diagnosis		68.5 ± 10.4	69.3 ± 9.8
Pathologic Stage	I	17	16
	II	13	15
	III	71	9
	IV	0	3
	Discrepancy	0	2
Subtype	ADC	49	19
	SCC	52	26

## Supplementary Materials

The following are available online at https://www.mdpi.com/2072-6694/12/12/3663/s1, Table S1: Differentiating features between adenocarcinomas and squamous cell carcinomas with respect to demographics, routine imaging and pathology, Figure S1: Box plots (**a**–**f**) comparing the six top-ranked pathomics features at different tile sizes (50 μm^2^, 33.3 μm^2^ and 25 μm^2^), between ADC (blue) to SCC (yellow). Box plots are presented for the CPTAC database, with no significant differences observed between tile sizes, Figure S2: Box plots (**a**–**f**) of the six top-ranked radiomics features comparing between CT manufacturers, i.e., Siemens (blue) to CMS (red). Box plots are presented for the NSCLC-R dataset, showing no significant differences between the manufacturer sub-groups, Figure S3: Heatmap visualizations for (**a**,**d**) Spearman’s rank correlation coefficients between pathologic cell density and radiologic intensity statistics (red = negative correlation, blue = positive correlation). (**a**,**c**) correspond to cases with <15 patches/study while (**b**,**d**) correspond to those with ≥15 patches/study.

## Abbreviations

The following abbreviations are used in this manuscript:

NSCLC	Non-small cell lung cancer
ADC	Adenocarcinoma
SCC	Squamous cell carcinoma
HV	Horizonal-vertical directions
D	Diagonal direction
SVM	Support vector machine
CT	Computer tomography
FDR	False discovery rate
