# Supplementary material for: Identifying Cross-Scale Associations between Radiomic and Pathomic Signatures of Non-Small Cell Lung Cancer Subtypes: Preliminary Results
Source: Cancers (Basel). 2020 Dec 7;12(12):3663. doi: 10.3390/cancers12123663 (PMC7762258; doi:10.3390/cancers12123663)
Supplement: Supplementary file 1 [file cancers-12-03663-s001.pdf]

Supplementary Material: Identifying Cross-Scale Associations Between Radiomic and Pathomic Signatures of Non-Small Cell Lung Cancer Subtypes: Preliminary Results

Charlems Alvarez-Jimenez, Alvaro A. Sandino, Prateek Prasanna, Amit Gupta, Satish E. Viswanath and Eduardo Romero

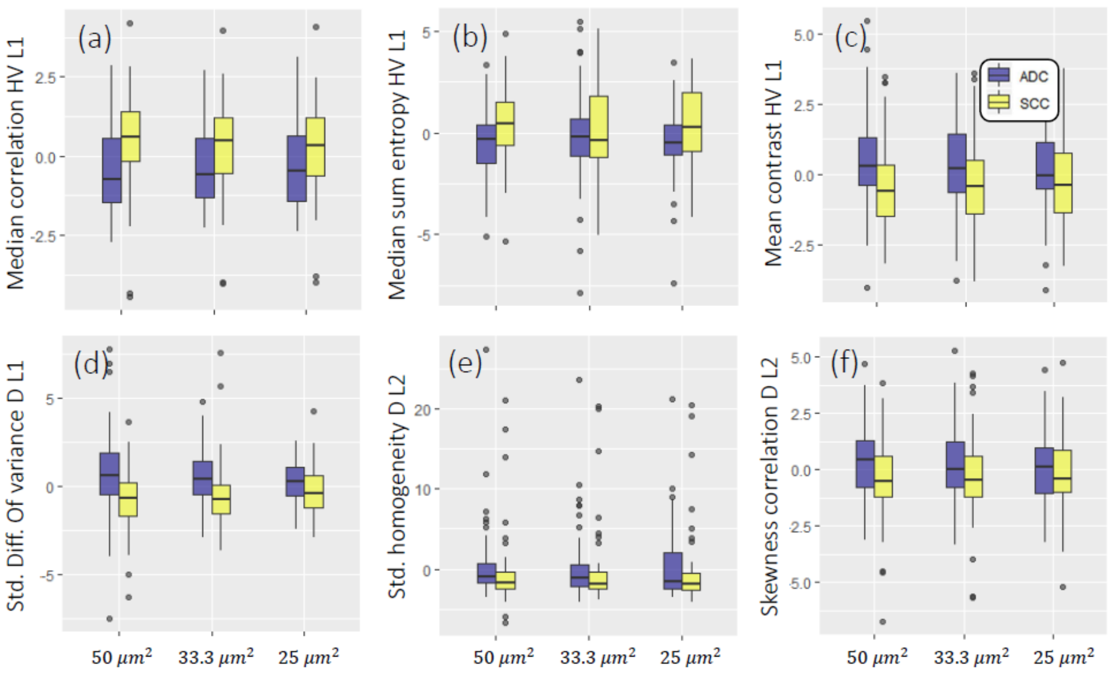

Figure S1. Box plots (**a**–**f**) comparing the six top-ranked pathomics features at different tile sizes (50 μm², 33.3 μm² and 25 μm²), between ADC (blue) and SCC (yellow). Box plots are presented for the CPTAC database, with no significant differences observed between tile sizes.

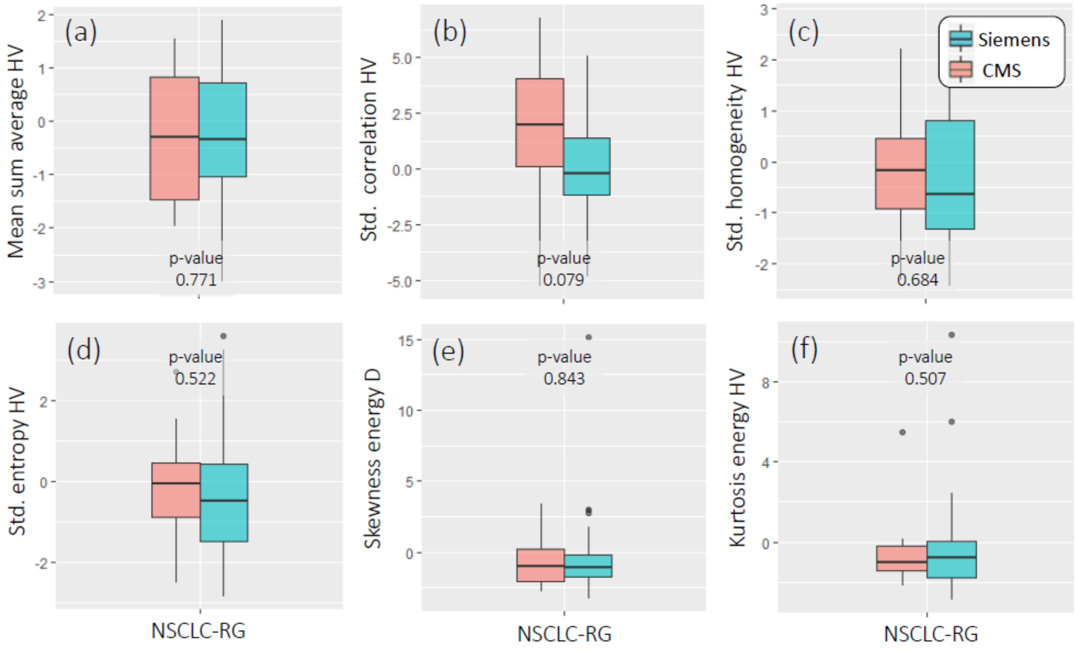

Figure S2. Box plots (**a**–**f**) of the six top-ranked radiomic features from different CT manufacturers, i.e., Siemens (blue) and CMS (red). Box plots are presented for the NSCLC-R dataset, showing no significant differences between the manufacturer sub-groups.

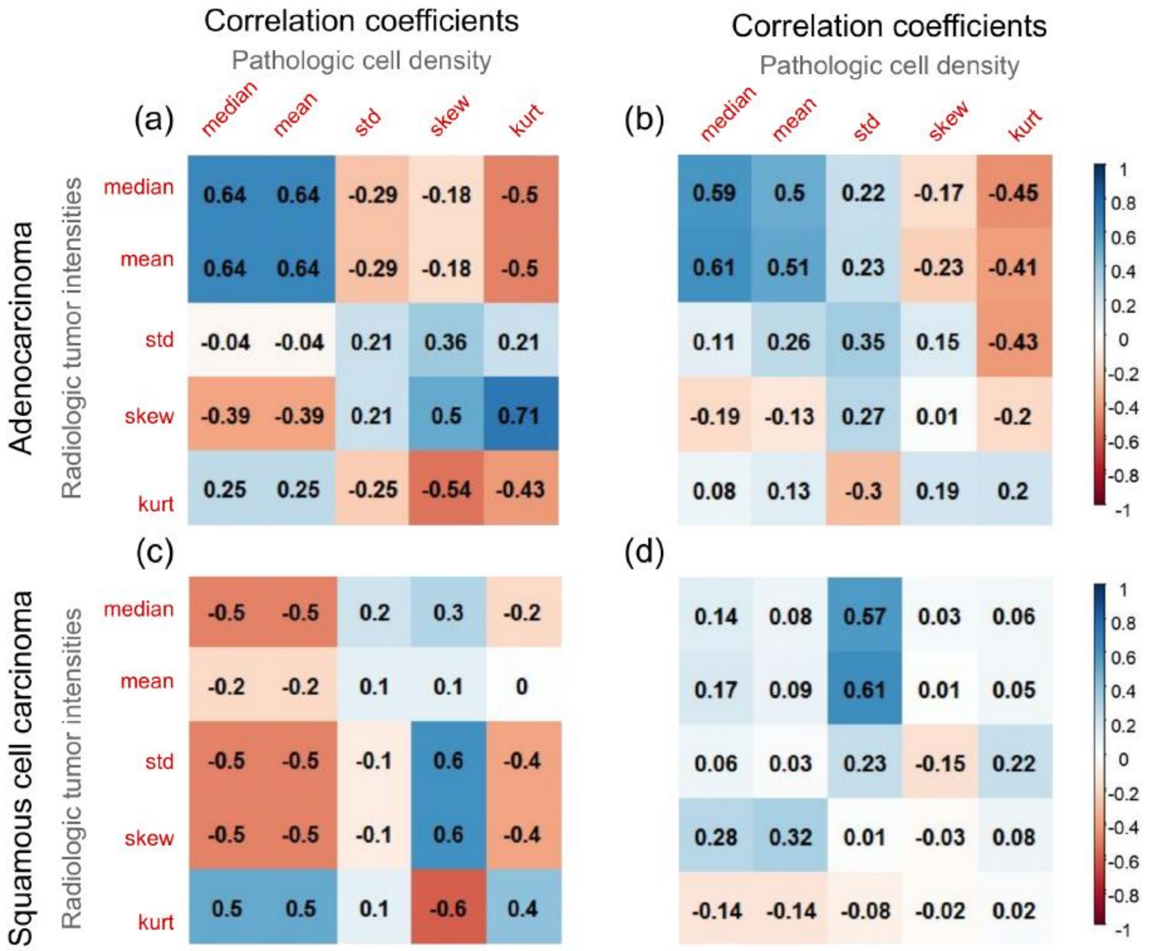

Figure S3. Heatmap visualizations for (**a**), (**d**) Spearman's rank correlation coefficients between. pathologic cell density and radiologic intensity statistics (red = negative correlation, blue = positive.

correlation). (**a**), (**c**) correspond to cases with < 15 patches/study while (**b**), (**d**) correspond to those with ≥15 patches/study.

Table S1. Differentiating features between adenocarcinomas and squamous cell carcinomas with. respect to demographics, routine imaging and pathology.

Characteristics	Adenocarcinomas	Squamous cell carcinoma
**Age** [1]	Relatively younger	Older
**Sex** [2]	Female predominance	Male predominance
**Smoking history** [1]	More common in non-smokers	More common in smokers
**Incidence** [3]	More common(50 % of NSCLC)	Less common (30 % of NSCLC)
**Behavior and Metastatic potential**	Less aggressive overall	More aggressive and relatively high metastatic potential
**Genomic alterations**	More common	Less common
**Treatment options and Prognosis**	More targeted therapies available with relatively better prognosis [6]	Less actionable mutations found with relatively worse prognosis
**Imaging** [4]:<br>**1. Location**<br>**2. Morphology**<br>**3. Margins**<br>**4. Air bronchograms**<br>**5. Post contrast enhancement**	1. More commonly peripheral<br>2. Ranging from ground glass to solid<br>3. Concave<br>4. More common<br>5. Generally homogenous	1. More commonly central<br>2. Solid with cavitation a frequent finding(as high as 82 percent) [7]<br>3. Lobulated<br>4. Rarely seen<br>5. Often heterogeneous
**Pathology** [5]:<br>**1. Typical microscopic appearance**<br>**2. Vascular bundle sign and the pleural indentation**<br>**3. Immunohistochemistry**	1. Varied appearance according to type but glandular differentiation is a hallmark<br>2. More common<br>3. Thyroid transcription factor-1 (TTF-1) and Napsin A positive in 85 percent cases. Cytokeratin (CK) 7 positive and CK 20 negative.	1. Keratinization and intercellular bridges with solid nested growth pattern.<br>2. Less common<br>3. TTF-1 negative, but positive for CK 5, 6 and P63 or P40

References

1. Yue, J.Y.; Chen, J.; Zhou, F.M.; Hu, Y.; Li, M.X.; Wu, Q.W.; Han, D.M. CT-pathologic correlation in lung adenocarcinoma and squamous cell carcinoma. *Medicine* **2018**, *97*, e13362, doi:10.1097/md.0000000000013362.
2. Devesa, S.S.; Bray, F.; Vizcaino, A.P.; Parkin, D.M. International lung cancer trends by histologic type: Male: female differences diminishing and adenocarcinoma rates rising. *Int. J. Cancer* **2005**, *117*, 294–299.
3. Wang, Z.; Li, M.; Huang, Y.; Ma, L.; Zhu, H.; Kong, L.; Yu, J. Clinical and radiological characteristics of central pulmonary adenocarcinoma: A comparison with central squamous cell carcinoma and small cell lung cancer and the impact on treatment response. *OncoTargets Ther.* **2018**, *11*, 2509.
4. Santos, M.K.; Muley, T.; Warth, A.; de Paula, W.D.; Lederlin, M.; Schnabel, P.A.; Schlemmer, H.P.; Kauczor, H.U.; Heussel, C.P.; Puderbach, M. Morphological computed tomography features of surgically resectable pulmonary squamous cell carcinomas: Impact on prognosis and comparison with adenocarcinomas. *Eur. J. Radiol.* **2014**, *83*, 1275–1281, doi:10.1016/j.ejrad.2014.04.019.
5. Zheng, M. Classification and Pathology of Lung Cancer. *Surg. Oncol. Clin. N. Am.* **2016**, *25*, 447–468, doi:10.1016/j.soc.2016.02.003.
6. Asamura, H.; Goya, T.; Koshiishi, Y.; Sohara, Y.; Eguchi, K.; Mori, M.; Nakanishi, Y.; Tsuchiya, R.; Shimokata, K.; Inoue, H.; et al. A Japanese Lung Cancer Registry study: Prognosis of 13,010 resected lung cancers. *J. Thorac. Oncol.* **2008**, *3*, 46–52.
7. Hollings, N.; Shaw, P. Diagnostic imaging of lung cancer. *Eur. Respir. J.* **2002**, *19*, 722–742.

Publisher's Note: MDPI stays neutral with regard to jurisdictional claims in published maps and institutional affiliations.

© 2020 by the authors. Licensee MDPI, Basel, Switzerland. This article is an open access article distributed under the terms and conditions of the Creative Commons Attribution (CC BY) license (http://creativecommons.org/licenses/by/4.0/).
